# Taxonomic review, palaeoecological, and palaeobiogeographical significances of Campanian Tethyan oysters from the North Eastern Desert, Egypt

**DOI:** 10.1038/s41598-024-63379-z

**Published:** 2024-06-12

**Authors:** El Sayed M. Moneer, Youssef S. Bazeen, Islam El-Sheikh, Ahmed Samir

**Affiliations:** 1 Geology Department, Faculty of Science, Al-Azhar University, Nasr City, Cairo Egypt; 2 Geology Department, Faculty of Science, Al-Azhar University, Assiut Branch, PO. Box: 71524, Assiut, Egypt

**Keywords:** Campanian, Oysters, Egypt, Tethyan affinity, Palaeoecological interpretations, Geology, Palaeontology

## Abstract

The Late Cretaceous was a time of high eustatic sea level that enabled extensive epicontinental seaways and carbonate platforms across the Tethyan Realm, providing favorable habitats for oyster communities to flourish. This study focuses on the Campanian Tethyan oysters from the North Eastern Desert of Egypt regarding taxonomy, palaeoecology, and palaeobiogeography. Three oyster species, *Nicaisolopha nicaisei* (Coquand, 1862), *Pycnodonte* (*Phygraea*) *vesicularis* (Lamarck, 1806), and *Ambigostrea bretoni* (Thomas and Peron, 1891), were identified from the Campanian succession in two studied sections. The sampled specimens of the genus *Nicaisolopha* have undergone a systematic palaeontological revision. As a result, *N. tissoti* (Thomas and Peron, 1891) is considered herein a junior synonym of *N. nicaisei* (Coquand, 1862). Palaeobiogeographically, the likely primary migration pattern of the studied oysters suggests an east–west trend along the Southern Tethys margin. All identified oysters in this study exhibit a Tethyan affinity and are primarily abundant in two main provinces: the Southern Tethys and the Western Tethys. The macrofaunal contents are categorized into two fossil associations: the *Nicaisolopha nicaisei* association of the middle-late Campanian age and the *Pycnodonte vesicularis* association of the late Campanian age. These macrofaunal associations indicate a deepening trend during the middle-late Campanian age, suggesting a transition from shallow inner neritic to middle neritic environments. Additionally, it is observed that Pycnodonteinae tend to grow larger under eutrophic conditions, low-energy environments, and nutrient-rich waters with high carbonate contents.

## Introduction

The major Late Cretaceous sea-level rise^1,2^ fostered the expansion of broad, shallow epicontinental sea and carbonate platforms over the vast Tethyan Realm. These environmental changes provided favorable ecological niches for oysters to proliferate and diversify within the abundant shallow marine ecosystems of the region^3^. During the Campanian, a broad, shallow epicontinental sea inundated southern Tethyan platforms, creating ideal habitat conditions for oyster communities to thrive. The warm, clear waters and abundant nutrients further supported the expansion of extensive oyster beds and reefs. Consequently, the Campanian sedimentary strata in Egypt preserve abundant and moderately diverse well-preserved oyster fossils, signaling the prominence of these bivalves in the Tethyan marine ecosystems. Based on shell morphology and anatomical features, three prominent oyster groups were distinguished in the region: the Pycnodonteinae, Flemingostreinae, and Liostreinae^4–6^.

The Campanian oyster in the North Eastern Desert has been studied by a few authors (e.g., Greco^7^, Fourtau^8^, Malchus^4^, and Zakhera et al.^9^), and most of these studies focused on the systematic paleontology of these oysters. Oysters, as sedentary filter-feeders closely linked to ambient environmental conditions, play a crucial role as indicators when it comes to reconstructing palaeoenvironmental parameters, habitat conditions, and biotic responses to shifts in ecosystems. Furthermore, analyzing the biogeography of a widely dispersed group like this can shed light on species dispersal patterns and factors controlling their geographical distribution. Therefore, a comprehensive investigation of the Campanian Tethyan oysters, encompassing their morphological variation, palaeoecology, and palaeobiogeography across the North African Epicontinental Sea, is highly justified. Hence, this paper focuses on the taxonomy, palaeoecology, and palaeobiogeography of Tethyan Campanian oysters from the Egyptian North Eastern Desert. A comprehensive examination of these critical aspects will further provide deeper insights into the palaeoenvironments and biotic interactions that have influenced the development of these pivotal Tethyan bivalve communities. The present study highlights the necessity of re-evaluating the taxonomic characteristics of *Pycnodonte* (*Phygraea*) *vesicularis* (Lamarck, 1806) from the Danian beds of the Egyptian Western Desert, due to the observed differences between these specimens and those from the Upper Cretaceous successions.

## Geologic setting and lithostratigraphy

This study focuses on analyzing the Campanian macrofaunal associations of the northern region of Egypt's Eastern Desert. This area was located along the Southern Tethys Ocean's passive continental margin during the Late Cretaceous. Over time, the shallow epicontinental sea in this region gradually became deeper as it moved northward, eventually forming the Neo-Tethys Ocean basin^10–13^. The Syrian Arc Fold Belt developed along the northern passive margin of the African-Arabian plate due to the convergence of Africa and Eurasia, starting in the Turonian^14^. This led to the inversion and uplift of fault-bounded Cretaceous rift basins that formed during the earlier Jurassic-Cretaceous extension^11,15^. The Late Cretaceous epoch was characterized by a greenhouse climate, which experienced fluctuations between periods of elevated warmth and cooler temperatures^16,17^. During the Campanian, the Southern Tethys margin exhibits the characteristics of a warm seasonal greenhouse climate^17^.

**Figure 1:**
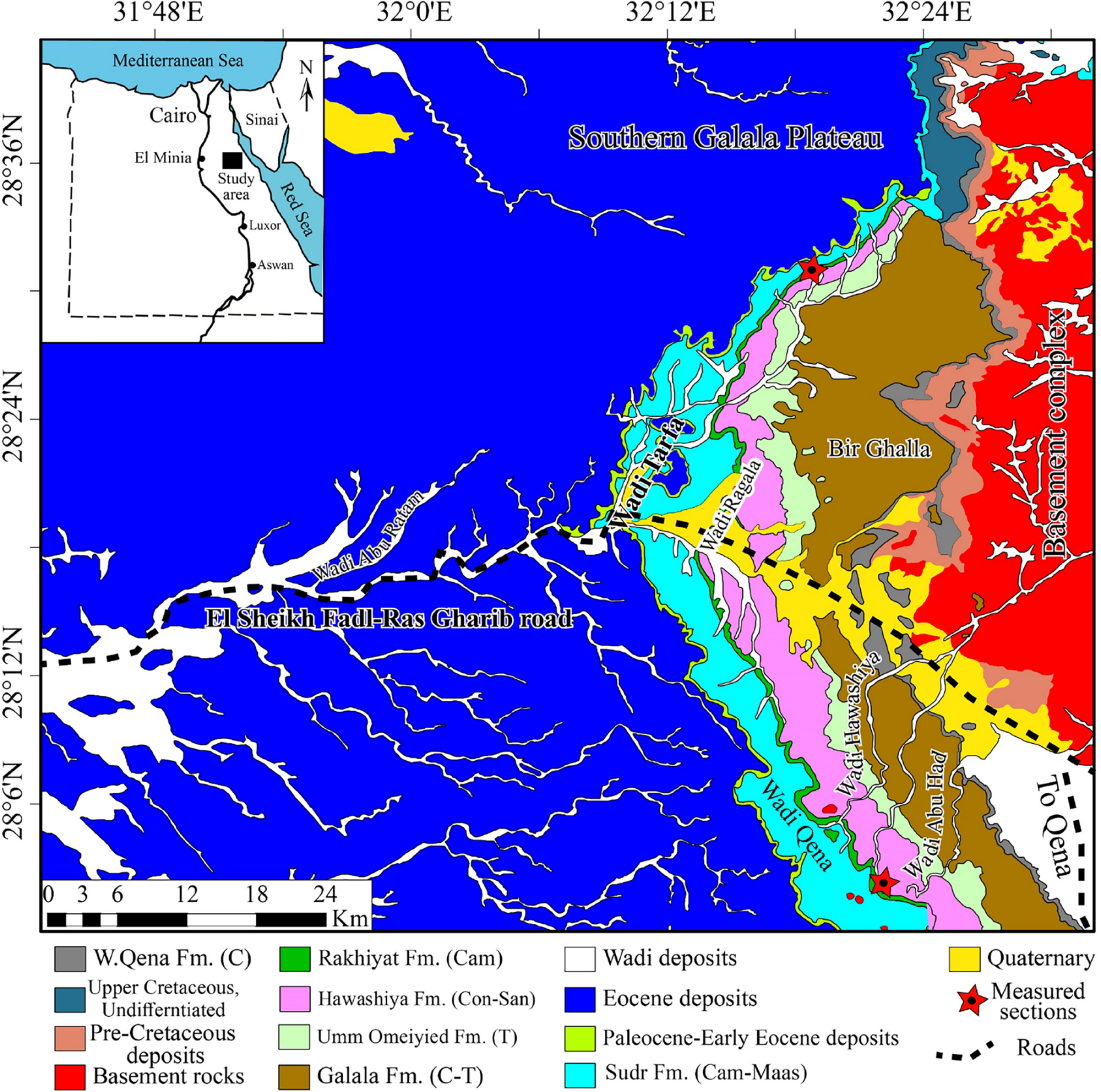
Geological map of the North Eastern Desert with the location of the studied sections (reprinted after Bazeen et al.^10^ with permission from Elsevier). This geological map was modified from the geological map of Egypt, Beni Suef sheet- *NH36SW* (after Klitszch et al.^18^).

**Figure 2:**
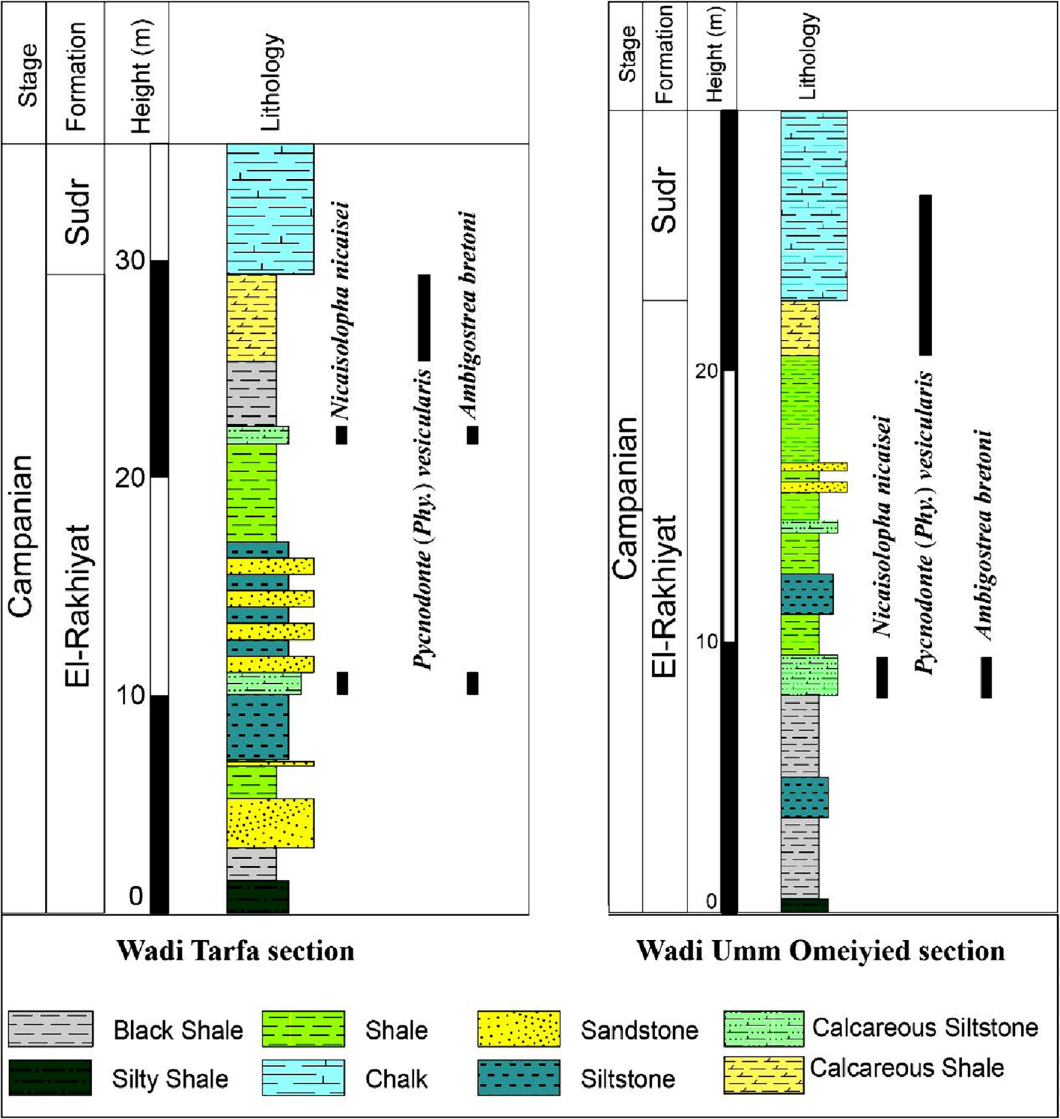
Stratigraphic successions of the studied Wadi Tarfa and Wadi Umm Omeiyied sections, with the distribution of the identified oysters.

We examined oyster fossils collected from two stratigraphic sections, the Wadi Tarfa and Wadi Umm Omeiyied sections, measured in the North Eastern Desert of Egypt (Fig. 1). In these sections, the Campanian deposits are represented by the El-Rakhiyat Formation and the lower part of the Sudr Formation (Fig. 2).

**Figure 3:**
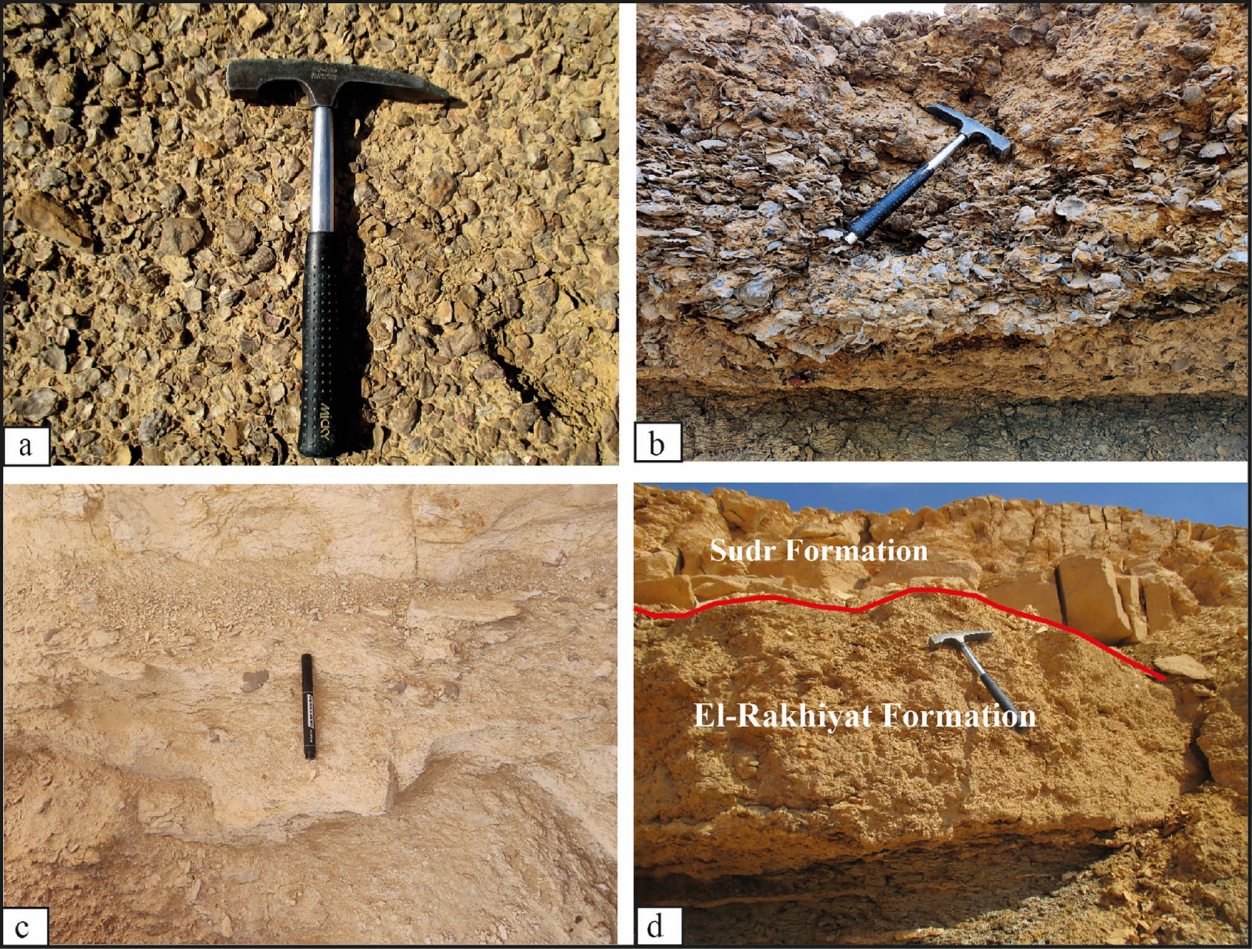
Filed photographs of the studied Wadi Tarfa and Wadi Umm Omeiyied sections: (**a**,**b**) The lower part of the El-Rakhiyat Formation enriched with *Nicaisolopha nicaisei* concentrations in the Wadi Tarfa (**a**) and Wadi Umm Omeiyied (**b**) sections. (**c**) The lower part of the Sudr Formation with *Pycnodonte* (*Phygraea*) *vesicularis*. (**d**) The unconformity boundary between the El-Rakhiyat and El Sudr formations in the Wadi Tarfa section.

The El-Rakhiyat Formation appears to be a laterally equivalent lithostratigraphic unit to the Duwi Formation, sharing a similar middle to late Campanian age range. The El-Rakhiyat Formation was first named by Hendriks^19^, with the type section located near the El-Rakhiyat village in the central Eastern Desert. It is characterized by grey to black shales with intermittent highly fossiliferous marly siltstone beds. The fissile shale is locally silty, sandy, phosphatic, ferruginous, and glauconitic. The formation reaches thicknesses between 20 and 30 m, with the lower part being very rich in *Nicaisolopha nicaisei* (Fig. 3a,b), while the upper part contains numerous small specimens of *Pycnodonte* (*Phygraea*) *vesicularis* (Fig. 3c). Based on the overlying Sudr Formation, which ranges from the latest Campanian to Maastrichtian^10^, the El-Rakhiyat Formation is also interpreted to be of middle to late Campanian age.

The Sudr Formation directly overlies the Rakhiyat Formation in an unconformable relationship (Fig. 3d). A distinct facies change can be observed between these two formations, with the Rakhiyat Formation characterized by clay-rich facies and the Sudr Formation exhibiting chalky facies. The investigated lower part of the Sudr Formation corresponds to the late Campanian age and falls within the planktic foraminifer *Globotruncanella havanensis* Zone^10^. It consists of pale yellow marly chalk and chalky limestone. The studied lower part of the Sudr Formation yields large-sized specimens of *Pycnodonte* (*Phygraea*) *vesicularis*.

## Materials and methods

**Figure 4:**
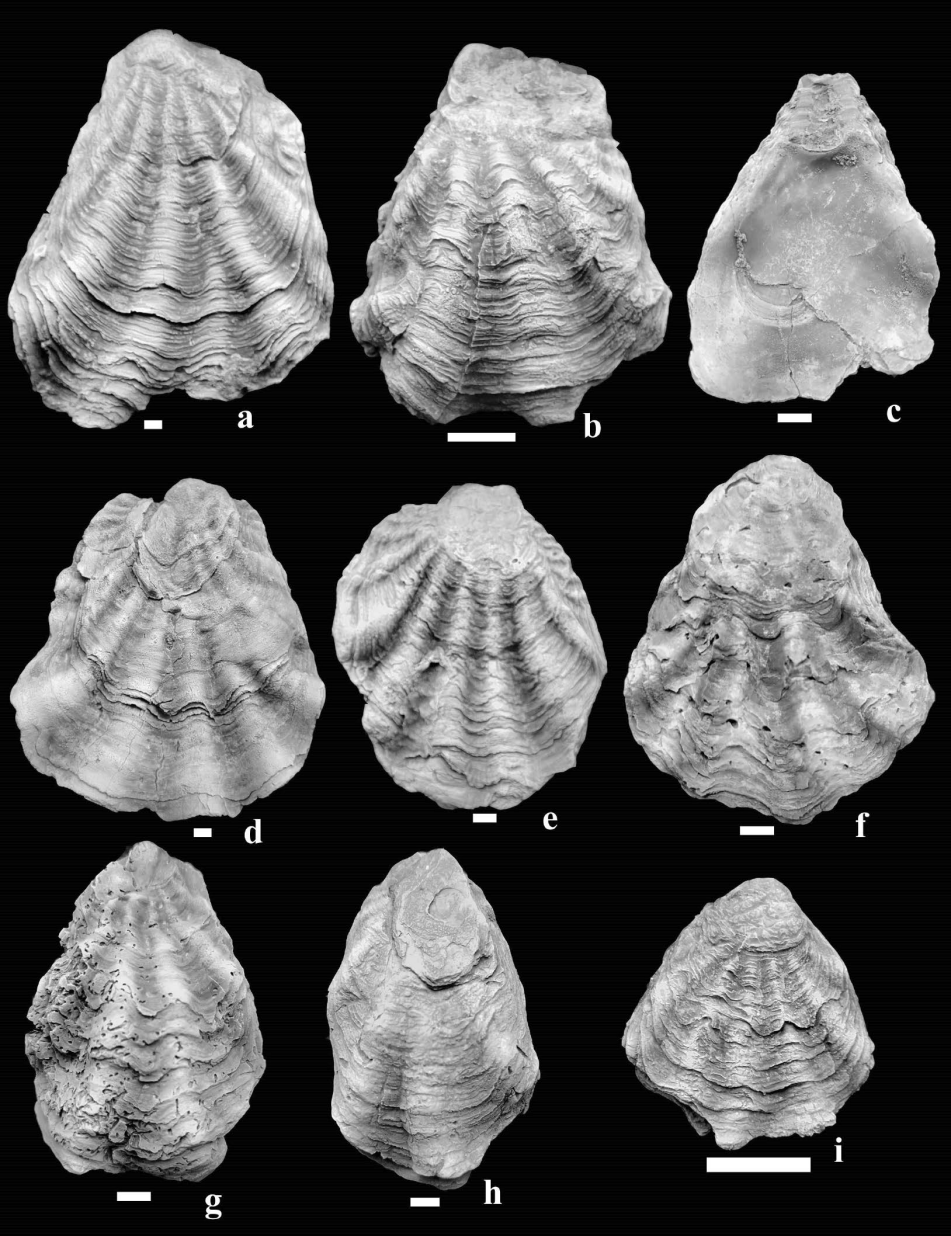
*Nicaisolopha nicaisei* (Coquand, 1862) from the Campanian successions of the Wadi Tarfa and Wadi Umm Omeiyied sections, in the North Eastern Desert, Egypt. (**a**,**d**) External views of right valves from the El-Rakhiyat Formation in the Wadi Tarfa section. (**b**,**e**,**h**,**i**) External views of left valves from the El-Rakhiyat Formation in the Wadi Tarfa section. (**c**) Internal view of the right valve from the El-Rakhiyat Formation in the Wadi Tarfa section. (**f**,**g**) External views of left valves from the El-Rakhiyat Formation in the Wadi Umm Omeiyied section. The bar scale is equal to 10 mm.

The oyster specimens analyzed in this study were collected from two stratigraphic sections located in the North Eastern Desert of Egypt: the Wadi Tarfa and Wadi Umm Omeiyied (Fig. 1). Surface picking was employed for fossil collection from the outcrop, and the specimens were subsequently subjected to washing and preparation in the laboratory for taxonomic identification. In total, 236 oyster specimens were collected. The collected oyster specimens were identified based on their external shell morphology and hinge structures following the systematic palaeontological descriptions by Stenzel^20^, Malchus^4^, Bieler et al.^21^ and Carter et al.^22^. Several well-preserved specimens from each species were measured for height (H), length (L), and thickness (T) using a Vernier Caliper in millimeters. These measurements facilitated the calculation of critical ratios (H/L, T/L, T/H) that serve as valuable diagnostic indicators for identifying oyster species. Representative macrofossil specimens were photographed and illustrated in Figs. 4 and 6. To better discern intricate shell morphologies, the imaged specimens underwent a coating procedure using ammonium chloride (NH_4_Cl), adhering to the methodology outlined by Feldmann^23^. All studied specimens were deposited in the Geological Museum of Al-Azhar University under the code AZGMCO followed by a running number.

## Systematic palaeontology

The studied Campanian successions in the North Eastern Desert are characterized by paucispecific macroinvertebrate communities containing only three oyster species. The classification and descriptive terminology of the studied oyster are primarily based on Stenzel^20^, with some modifications to incorporate more recent systematic revisions proposed by Malchus^4^, Bieler et al.^21^, and Carter et al.^22^, who suggested significant changes in the taxonomic categories above the genus level.

Family Ostreidae Rafinesque, 1815^24^.

Genus *Nicaisolopha* Vialov, 1936^25^.

*Nicaisolopha nicaisei* (Coquand, 1862)^26^.

Figure 4

1862 *Ostrea Nicaisei* Coquand^26^: p. 232, pl. 22, Figs. 5–7.

1869 *Ostrea Nicaisei* Coquand – Coquand^27^: p. 34, pl. 6, Figs. 1–17.

1891 *Ostrea Tissoti* Thomas and Peron in Peron^28^: p. 196, pl. 24, Figs. 1–7.

1903 *Alectryonia Tissoti* Thomas and Peron – Dacqué^29^: p. 365, pl. 34, Figs. 11–12.

1903 *Ostrea Tissoti* Thomas and Peron – Fourtau^8^: p. 639, pl. 24, Figs. 1–7.

1917 *Alectryonia Nicaisei* Coquand – Greco^7^: 116 (136), pl. 14 (13), Figs. 2, 3.

1917*Ostrea Tissoti* Thomas and Peron – Fourtau^30^: p. 54, pl. 5, Figs. 1–5.

1917 *Ostrea Nicaisei* Coquand – Fourtau^30^: p. 43, pl. 6, Figs. 1–4.

1966 *Ostrea nicaisei* Coquand – Willard^31^: p. 128, 130, pl. 12, Fig. 3; pl. 13, Fig. 1.

1987 *Nicaisolopha nicaisei* (Coquand) – Bandel et al.^32^: pl. 2, figs. Za, b.

1990 *Nicaisolopha nicaisei* (Coquand) – Malchus^4^: p. 174, pl. 19, Figs. 17 and 19; pl. 20, Figs. 1–8.

1990 *Nicaisolopha tissoti* (Thomas and Peron) – Malchus^4^: p.174, pl. 19, Figs. 7–16, 18.

1993 *Nicaisolopha nicaisei* (Coquand) – Aqrabawi^33^: p. 86, pl. 6, Figs. 1–5.

1999 *Nicaisolopha nicaisei* (Coquand) – Dhondt et al.^34^: pl. 1, Figs. 9, 10.

2001 *Cameleolopha* (*Hyotissocameleo*)* tissoti* (Thomas and Peron) – Zakhera et al.^9^: 85, Fig. 7.

2005 *Nicaisolopha nicaisei* (Coquand) – Dhondt and Jaillard^35^: p. 337, pl. 2, Figs. 3–5.

2006 *Nicaisolopha nicaisei* (Coquand) – El Qot^6^: p. 50, pl. 9, Figs. 3–5, 6.

2006 *Nicaisolopha tissoti* (Thomas and Peron) – El Qot^6^: p. 52, pl. 9, Figs. 7, 8; pl. 10, Figs. 1, 2.

**Materials:** Two hundred specimens were collected from the Campanian Rakhiyat Formation in the Wadi Umm Omeiyied and Wadi Tarfa sections, including 40 complete articulated specimens, 100 left valves, and 60 right valves.

### Measurements

**Table:** 

S.N.	H	L	T	H/L	T/L	T/H	S.N.	H	L	T	H/L	T/L	T/H
AZGMCO-1	56	41	12	1.4	0.3	0.2	AZGMCO-18	50	36.5	11.5	1.4	0.3	0.2
AZGMCO-2	55	43	15	1.3	0.3	0.3	AZGMCO-19	55	39	13.5	1.4	0.3	0.2
AZGMCO-3	53	39	9	1.3	0.2	0.2	AZGMCO-20	64.5	43.5	9	1.5	0.2	0.1
AZGMCO-4	62	45	13	1.4	0.3	0.2	AZGMCO-21	51	46	8	1.1	0.2	0.1
AZGMCO-5	59	40	13	1.5	0.3	0.2	AZGMCO-22	54	37	10	1.4	0.3	0.2
AZGMCO-6	52	42	10	1.2	0.2	0.2	AZGMCO-23	50	46	9	1.1	0.2	0.2
AZGMCO-7	57	49	11	1.2	0.2	0.2	AZGMCO-24	54	41.5	12	1.3	0.3	0.2
AZGMCO-8	53	39	14	1.3	0.3	0.3	AZGMCO-25	37	34	6	1.1	0.2	0.2
AZGMCO-9	61	49	11	1.2	0.2	0.2	AZGMCO-26	49	41	11	1.2	0.3	0.2
AZGMCO-10	75	49	16	1.5	0.3	0.2	AZGMCO-27	55	48	11.5	1.1	0.2	0.2
AZGMCO-11	54	45	9	1.2	0.2	0.2	AZGMCO-28	71.5	41	9.5	1.7	0.2	0.1
AZGMCO-12	69	52	10	1.3	0.2	0.1	AZGMCO-29	45	38	11	1.2	0.3	0.2
AZGMCO-13	63	48	11	1.3	0.2	0.2	AZGMCO-30	45.5	37.5	8	1.2	0.2	0.2
AZGMCO-14	63	43	10	1.5	0.2	0.1	AZGMCO-31	37.5	33	8	1.1	0.2	0.2
AZGMCO-15	50	46	12	1.1	0.3	0.2	AZGMCO-32	51.5	38	11	1.3	0.3	0.2
AZGMCO-16	58	41	16	1.4	0.4	0.3	AZGMCO-33	51	31	9.5	1.6	0.3	0.2
AZGMCO-17	56	45	13	1.2	0.3	0.2	AZGMCO-34	59	39	14	1.5	0.3	0.2

**Description:** Medium-sized, oval or subtriangular, inflated and thick-shelled; subequivalves with a slightly more convex left valve than the right one; attachment area highly variable from very small to large, sometimes limited to the apex of the ribs; initially with numerous rounded radial folds; adductor scars placed on the posterio-ventral margin or subcentrally and variable from kidney-shaped to weakly crescent-shaped; umbo small and approximately orthogyrate; ligament area long or inclined; left valve resilifer long and shallow with well-developed bourrelets, right valve resilifer flat; no chomata; right and left valves are ornamented with a few rounded-crested, not dichotomous, widen ventrally, wavy radial folds crossed by irregular, closely spaced, undulatory, slightly raised commarginal ribs, and separated by equal, rounded interspaces, ending at commissural folds; the radial folds of the left valve are more pronounced and robust when compared to those of the right valve.

**Remarks:** *Nicaisolopha nicaisei* exhibits significant variations in the number and size of its radial folds, the spacing of its large concentric lamellae, and the degree of convexity of its left valve. These variations have been observed by several authors, including Malchus^4^, who highlighted that distinguishing smaller specimens of *N. nicaisei* with irregular ribbing from *N. lyonsi* can be challenging. Additionally, some specimens exhibit sculptured characteristics similar to *N. tissoti*. Aqrabawi^33^ pointed out that young specimens of *N. nicaisei* may exhibit similarities to *N. tissoti*.

**Figure 5:**
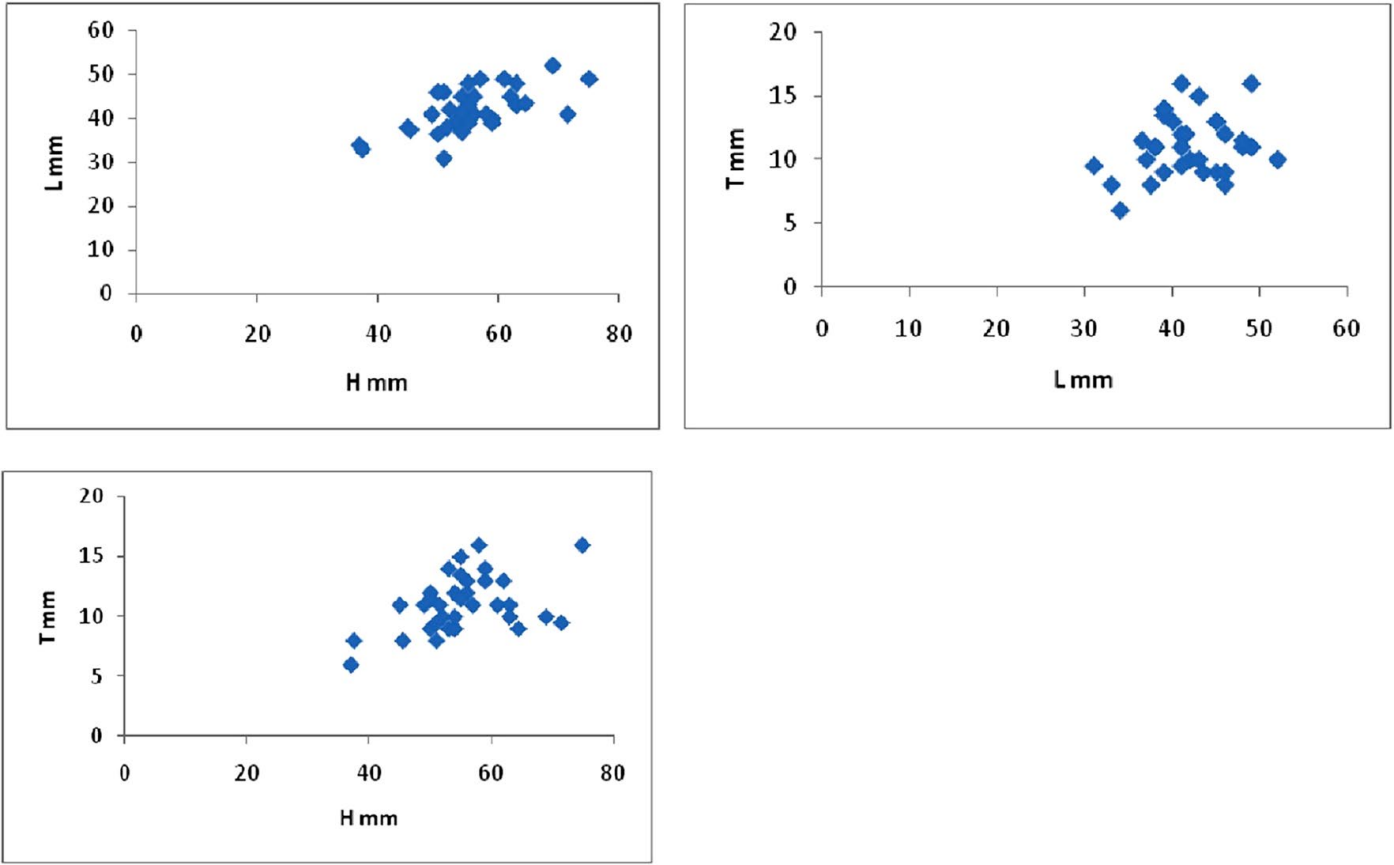
Biometric data of *Nicaisolopha nicaisei* (Coquand, 1862).

The collected specimens are characterized by a high degree of morphological variation, which can be categorized into two main stages: the early stage and the late stage, with intermediary forms linking the two. The late stage is characterized by moderate to large size, featuring a strongly convex left valve ornamented with wide undulating radial folds intersected by irregular and closely spaced commarginal ribs. This stage was initially designated as *Ostrea Nicaisei* by Coquand^26^ based on findings from the Campanian of Algeria. Thomas and Peron subsequently identified the early stage in 1891^28^, also derived from the same stratigraphic interval in Algeria, as *Ostrea tissoti*. It is characterized by its small to medium size, thin shells, the presence of faint, undulating, plicate radial ribs, and inconspicuous growth lamellae. In the present study, numerous specimens were collected, and transitional forms connecting these two stages were observed. Furthermore, the biometric data of this species demonstrates a diverse range of variations in terms of length, height, and width (Fig. 5). Consequently, *Nicaisolopha tissoti* (Thomas and Peron 1891)^28^ is considered a junior synonym of *Nicaisolopha nicaisei* (Coquand, 1862)^26^.

Family Gryphaeidae Vialov, 1936^25^.

Subfamily Pycnodonteinae Stenzel, 1959^36^.

Genus *Pycnodonte* Fischer de Waldheim, 1835^37^.

Subgenus *Phygraea* Vialov, 1936^25^.

*Pycnodonte* (*Phygraea*) *vesicularis* (Lamarck, 1806)^38^.

**Figure 6:**
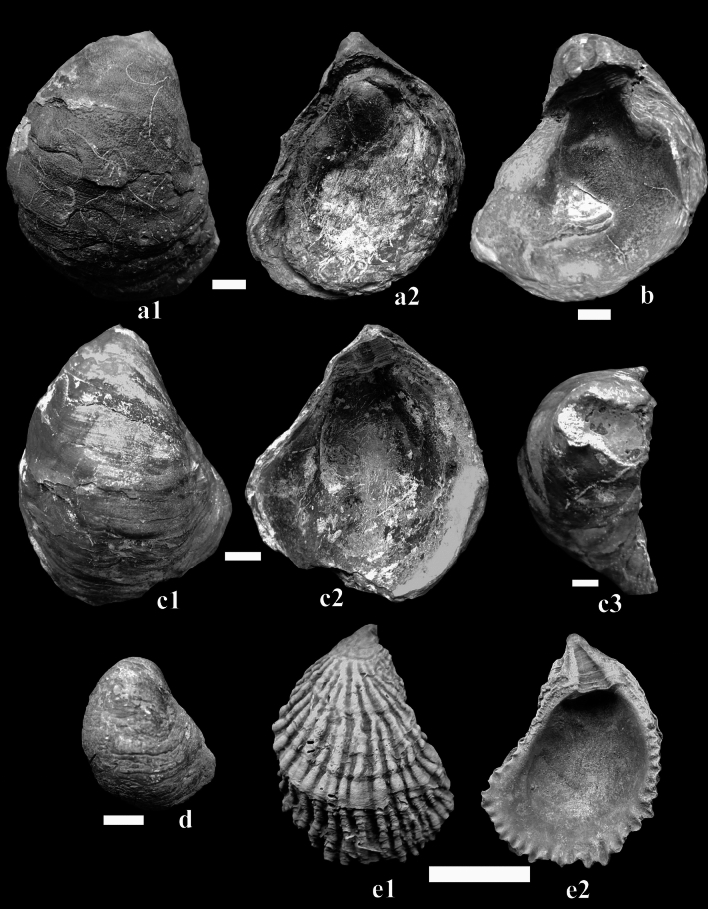
Oysters from the Campanian successions of the Wadi Tarfa and Wadi Umm Omeiyied sections, in the North Eastern Desert, Egypt. (**a1**,**a2**,**b**,**c1**,**c2**,**c3**,**d**) *Pycnodonte* (*Phygraea*) *vesicularis* (Lamarck, 1806) from the Campanian Sudr Formation. (**a1**,**c1**,**d**) External views of left valves. (**a2**) External view of right valve. (**b**,**c2**) Internal views of left valves. (**c3**) Dorsal view of the left valve. (**e1**,**e2**) *Ambigostrea bretoni* (Peron and Thomas, 1891) from the Campanian succession of the Wadi Tarfa section. (**e1**) External view of left valve. (**e2**) Internal view of left valve. The bar scale is equal to 10 mm.

Figure 6a1,a2,b,c1,c2,c3,d.

1806 *Ostrea vesicularis* Lamarck^38^: p. 160.

1809 *Ostrea vesicularis* Lamarck – Lamarck^39^: p. 375, pl. 22, Fig. 3.

1871*Gryphea vesicularis* (Lamarck) – Stoliczka^40^: p. 465, pl. 42, Figs. 2–4; pl. 43, Fig. 1; pl. 45, Figs. 7–12.

1912 *Pycnodonte vesicularis* Lamarck – Pervinquière^41^: p. 195.

1913* Ostrea vesicularis* Lamarck – Woods^42^: p. 360, pl. 15, Figs. 4–7.

1917 *Ostrea vesicularis* Lamarck – Fourtau^30^: p. 55.

1918 *Pycnodonta vesicularis* Lamarck – Greco^43^: p. 110 (130), pl. 13 (12), Figs. 1–5.

1962 *Pycnodonte vesicularis* (Lamarck) – Abbass^44^: p. 71, pl. 10, Figs. 1, 2.

1972 *Pycnodonte* (*Pycnodonte*)* vesicularis vesicularis* (Lamarck) – Freneix^45^: p. 105, pl. 10, Figs. 5–7.

1977 *Pycnodonte* (*Phygraea*)* vesicularis* (Lamarck) – Pugaczewska^46^: p. 191, pl. 13, Figs. 1–3.

1986 *Pycnodonte* (*Phygraea*)* vesiculare* (Lamarck) – Abdel-Gawad^47^: p. 162, pl. 38, Fig. 5.

1990 *Pycnodonte* (*Phygraea*)* vesiculare* (Lamarck) – Malchus^4^: p. 146, pl. 2, Figs. 8–10; pl. 3, Figs. 1–3, 5. (with detailed synonymy).

1991 *Pycnodonte* vesiculare (Lamarck) – Darragh and Kendrick^48^: p. 28, Figs. 5A–N, 6A–L, 7A–C.

1993 *Pycnodonte* (*Phygraea*)* vesiculare* (Lamarck) – Aqrabawi^33^: p. 80, pl. 5, Fig. 3.

1993 *Pycnodonte* (*Phygraea*) *vesicularis* (Lamarck) – Dhondt^49^: p. 242.

1994* Pycnodonte* (*Phygraea*)* vesiculare* (Lamarck) – Malchus et al.^50^: p. 125, pl. 3, Figs. 1, 3, 5; pl. 6, Figs. 1–12.

1995*Pycnodonte* (*Phygraea*)* vesicularis* (Lamarck) – Strougo^51^: p. 10, Fig. 3 (9–10).

2006*Pycnodonte* (*Phygraea*)* vesicularis vesicularis* (Lamarck) – El Qot^6^: p. 39, pl. 5, Fig. 9.

2013*Pycnodonte* (*Phygraea*)* vesicularis vesicularis* (Lamarck) – El Qot et al.^52^: p. 179, pl. 2, Figs. 1, 2.

2014* Phygraea* (*Phygraea*)* vesicularis* (Lamarck) – Jaitly et al.^53^: p.42, Fig. 2A.

2014 *Phygraea* (*Phygraea*)* vesicularis* (Lamarck) – Brezina et al.^54^: Fig. 5.

2017 *Phygraea* (*Phygraea*)* vesicularis* (Lamarck) – Brezina et al.^52^: Figs. 1–4.

**Materials:** Three complete and two incomplete left valves were collected from the upper Campanian Rakhiyat Formation in the Wadi Tarfa section. Additionally, two complete articulated specimens, along with five complete left valves and ten incomplete left valves, were collected from the upper Campanian Sudr Formation in the Wadi Umm Omeiyied section.

### Measurements

**Table:** 

S.N	H	L	T	H/L	T/L	T/H	S.N	H	L	T	H/L	T/L	T/H
AZGMCO-35	73	56	32	1.3	0.6	0.4	AZGMCO-39	68	56	33	1.2	0.6	0.5
AZGMCO-36	70	57	30	1.2	0.5	0.4	AZGMCO-40	70	60	30	1.2	0.5	0.4
AZGMCO-37	85	60	42	1.4	0.7	0.5	AZGMCO-41	62	53	30	1.2	0.6	0.5
AZGMCO-38	56	47	29	1.2	0.6	0.5	AZGMCO-42	53	40	24	1.3	0.6	0.4

**Description:** Medium to large-sized, oval to obliquely triangular, slightly higher than long, moderately thick-shelled; inequivalves with the left valve larger and strongly convex, while the right valve is small and flat or slightly concave; posterior-dorsal margin concave and geniculate; umbo less incurved, prominent and orthogyrate to slightly opisthogyrate; deep umbonal cavity; well-developed posterior sulcus; attachment area variable in size from small to absent; ligament area obliquely triangular, often longer than high; adductor muscle scar situated near the posterior margin or subcentral, and with an oval to subrounded shape; concentric growth lines, separated by wide interspaces, covered the outer shell.

**Remarks:** Strougo^51^ and Hewaidy et al.^55^ have documented the occurrence of this species in the early Paleocene of Egypt. They concluded that the specimens from the Paleocene deposits exhibit more pronounced commarginal ribs than those found in the Upper Cretaceous. However, these Paleocene-recorded specimens need to be reexamined due to the significant differences between these samples and those of the Upper Cretaceous deposits.

Subfamily Flemingostreinae Stenzel, 1971^20^.

Tribe Ambigostreini Malchus, 1990^4^.

Genus *Ambigostrea* Malchus, 1990^4^.

*Ambigostrea bretoni* (Thomas andPeron, 1891)^28^.

Figure 6e1,e2.

1891*Ostrea Bretmi* Thomas and Peron in Peron^28^: p. 197, pl. 25, Figs. 37–39.

1990 *Ambigostrea bretoni* (Peron and Thomas) – Malchus^4^: p. 179, pl. 21, Figs. 13–25.

2006 *Ambigostrea bretoni* (Peron and Thomas) – El Qot^6^: p. 54, pl. 10, Figs. 3, 4.

**Materials:** Two complete and four incomplete left valves were collected from the Campanian Rakhiyat Formation in the Wadi Umm Omeiyied section.

### Measurements

**Table:** 

S.N	H	L	T	H/L	T/L	T/H	S.N	H	L	T	H/L	T/L	T/H
AZGMCO-43	22	17	7	1.3	0.4	0.3	AZGMCO-44	26	20	8	1.3	0.4	0.3

**Description:** Small, oval to triangular, thin-shelled; anterior margin convex, posterior margin concave; left valve inflated, sometimes keeled; right valve flat or concave; umbo sharp and slightly inclined; adductor muscle placed postero-ventral to sub central of the shell; attachment area relatively small, sometimes punctiform; ligament area sharp, highly-triangular, usually straight or slightly inclined; deep resilifer, flanked by flat bourrelets; umbonal cavity weak or not developed; ornamentation consists of irregular fine radial ribs that bifurcate near the ventral margin.

**Remarks:** According to Malchus^4^ *Ambigostrea dominici* Malchus, 1990^4^ from the Cenomanian of the Western Desert resembles *Ambigostrea bretoni* (Thomas and Peron, 1891)^28^, in terms of its small pointed apex and fine radial ribbing. However, *Ambigostrea bretoni* distinguishes itself by being larger, relatively flatter, slightly thicker-shelled, and having distinctive edge grooves.

## Palaeoecology and palaeoenvironment

The Campanian stage of the Late Cretaceous period was characterized by a significant transgression, leading to elevated sea levels that inundated continental interiors and formed extensive epicontinental seas. This transgression was a global phenomenon^55,56^, with North Africa, including Egypt, experiencing the emergence of a shallow marine embayment along the Southern Tethys margin. During this period, the region underwent significant transgression, with marine facies deposition reaching as far south as the Taoudeni-Iullemmeden-Chad-Al Kufra-Upper Egypt basins^57^. Therefore, interpreting Egyptian oyster species provides crucial palaeoecological evidence that aids in characterizing the Campanian settings of the Southern Tethys as far-field reflections of climatic and tectono-eustatic drivers. Their presence is linked to high relative sea levels, facilitating the northward spillover of Tethyan waters along the northern African coastline. The studied faunal contents can be classified into two main fossil associations: the *Nicaisolopha nicaisei* association and the *Pycnodonte vesicularis* association**.**

### *Nicaisolopha nicaisei* association

This association is recorded from the lower part of the Campanian Rakhiyat Formation in the Wadi Umm Omeiyied and Wadi Tarfa sections. It forms an oyster bank and is found within two ledges of calcareous siltstone in the Wadi Tarfa section and one ledge of calcareous siltstone in the Wadi Umm Omeiyied section. In the Wadi Tarfa section, the lower oyster bank has an approximate thickness of one meter, while the upper one measures approximately 80 cm. In the Wadi Umm Omeiyied section, the oyster bank has a thickness of about 1.5 m. The *Nicaisolopha nicaisei* association comprises two species: *Nicaisolopha nicaisei* (Coquand, 1862), and *Ambigostrea bretoni*. *Nicaisolopha* is a stationary epifaunal oyster characterized by prominent ribbing, and it is adapted for suspension feeding in normal marine salinity conditions. Attachment scars on the left valves exhibit high variability, ranging from very small to large, sometimes limited to the rib apex. The presence of attachment scars on some left valves suggests that the oyster remained attached to hard objects on the sea floor throughout its life^58^. The dominance of epifaunal organisms in this association indicates the predominance of firm, coarse-grained stable substrates^59^. Moreover, the prevalence of suspension feeders suggests an environment primarily influenced by a single source of nutrients, where food detritus is only available in the water.

**Figure 7:**
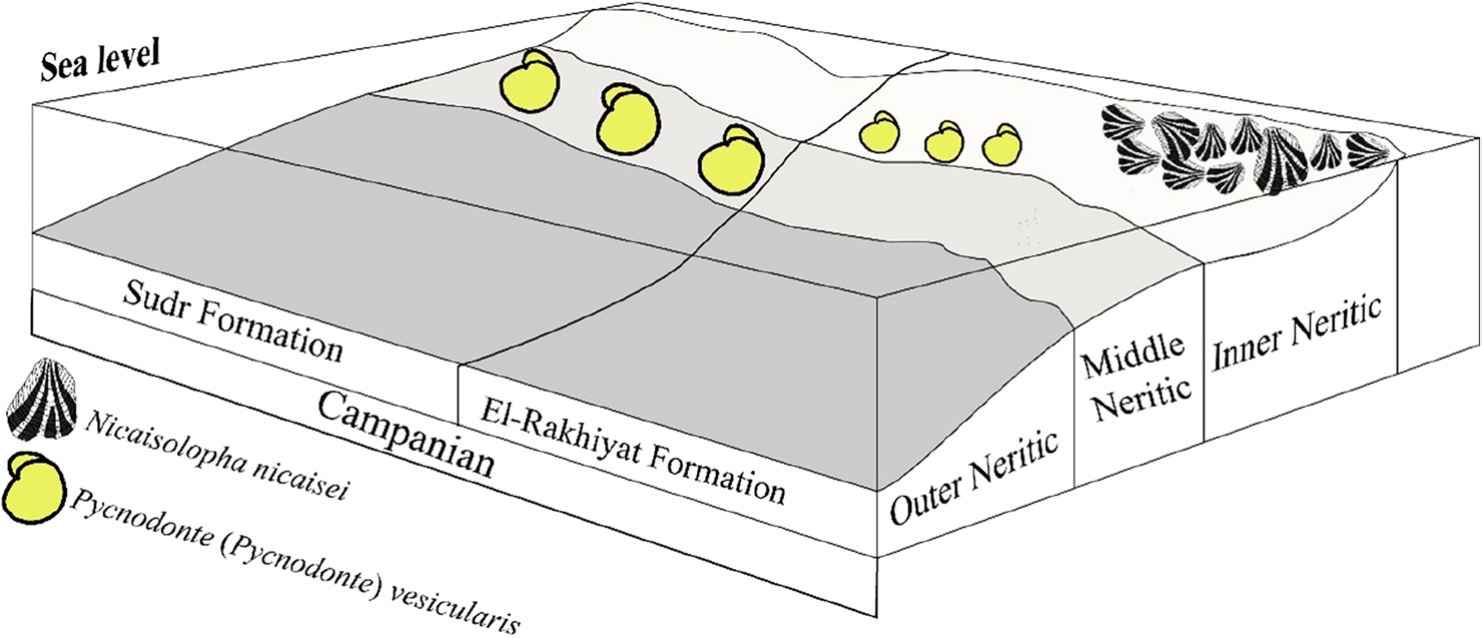
A palaeoenvironmental schematic diagram depicting the temporal and palaeoecological contexts of *Nicaisolopha nicaisei* association and *Pycnodonte vesicularis* association.

The *Nicaisolopha* fossil bank exhibits a notable level of regularity at its base, with approximately 80% of individuals found articulated and around 30% remaining in their original life position. Additionally, the specimens display a high degree of preservation with low levels of fragmentation, suggesting relatively short-distance transportation under a low-energy regime (parautochthonous association)^60^. The prevalence of articulated individuals indicates evidence of a significant mortality event, implying that these organisms were abruptly buried in their habitat by a sudden influx of sediment. This is also supported by the limited occurrence of perforations on the left and right valves, suggesting swift burial in areas with relatively high sedimentation rates. The oyster bank is situated near the base of siliciclastic beds consisting of siltstone, sandy shale, greenish shale, and black shale (Fig. 2). Therefore, based on the palaeontological and sedimentological data, the environmental interpretation suggests that this association thrived under normal marine conditions. It was characterized by low-energy conditions, well-oxygenated water, relatively high sedimentation rates, and a shallow inner neritic environment (Fig. 7) with hard substrates.

### *Pycnodonte vesicularis* association

This monospecific association was established based on a bulk-sample comprising 25 collected specimens of *Pycnodonte vesicularis*. These specimens were scattered (dispersed packing) in the uppermost part of the Rakhiyat Formation and the lowermost part of the Sudr Formation. The uppermost part of the Rakhiyat Formation primarily consists of grey fissile shale, intermittently interspersed with marly and calcareous shale bands. Nevertheless, the lowermost part of the Sudr Formation is composed of pale brown chalky limestone (Fig. 3c). While most oysters adapt well to various shallow-water environments, the Pycnodonteinae species likely inhabited deeper waters within the continental shelf facies, at depths exceeding 15 meters^31^. Such a preference implies a low sedimentation rate and indicates that these oysters inhabited muddy bottoms, which is indicative of increasingly calm water conditions^3,34,61^.

The studied species is characterized by a strongly convex left valve and a flat to slightly concave right valve. Additionally, most collected specimens lack attachment scars on the left valve. The asymmetrical and inequivalve shape of this species and the absence of attachment scars indicate that this species predominantly existed as a free-lying individual under low-energy conditions and on soft substrates^3,34,60,61^. However, only a few specimens exhibit attachment scars (Fig. 6c3), implying that only a minority of individuals were able to attach themselves to solid substrates on the ocean floor throughout their adult lifespan.

**Figure 8:**
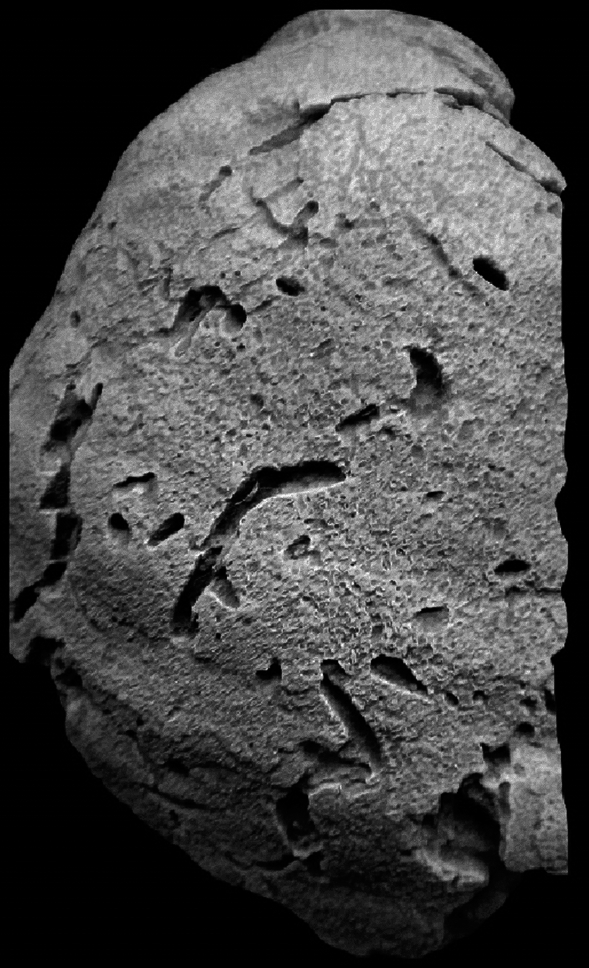
*Maeandropolydora* isp. on the external left valve of *Pycnodonte* (*Phygraea*) *vesicularis* from the Campanian El-Rakhiyat Formation in the Wadi Tarfa section.

The specimens of *Pycnodonte vesicularis* collected from the Rakhiyat Formation differ from those collected from the Sudr Formation in terms of their smaller size, thinner shell, and presence of bioerosion. These variations provide valuable insights into the contrasting palaeoecological and palaeoenvironmental conditions prevalent in the uppermost part of the Rakhiyat Formation and the lowermost part of the Sudr Formation. The bioerosion of the specimens collected from the Rakhiyat Formation can be attributed to the ichnogenus *Maeandropolydora* (Fig. 8). This trace fossil is known to be produced by suspension-feeding spionid polychaetes^54,62^. Moreover, the reduced incidence of perforations penetrating the valve interiors suggests that the organisms likely experienced bioerosion activity throughout their lifespan. This indicated that the relationship between *P*. (*Ph*.) *vesicularis* and polychaetes may be classified as commensalism^61^. Consequently, the uppermost portion of the Rakhiyat Formation was deposited in a deep inner neritic environment (Fig. 7) characterized by normal marine salinity, low rates of sedimentation, and soft substrates.

Based on foraminiferal data^10^, the lowermost portion of the Sudr Formation is interpreted to have been deposited in a middle neritic setting (Fig. 7), characterized by eutrophic conditions and fluctuating oxygenation levels. This portion also displays features of calm water conditions and nutrient-rich waters with high carbonate content. Moreover, the presence of scleobiont traces on the collected *Pycnodonte vesicularis* from the lower Sudr Formation (Fig. 6a,b) suggests the existence of predators within this community. The environmental conditions in the lower Sudr Formation may have facilitated the development of the comparatively large-sized *Pycnodonte vesicularis* oysters (Fig. 6a1,a2,b,c1,c2,c3) in comparison to specimens obtained from the underlying Rakhiyat Formation (Fig. 6d).

## Palaeobiogeography

In the present study, three Campanian oyster species were recorded from the North Eastern Desert. These include *Pycnodonte* (*Phygraea*) *vesicularis*, *Nicaisolopha nicaisei*, and *Ambigostrea bretoni*. It is worth noting that *Ambigostrea bretoni* may be restricted to the Campanian of the Southern Tethys margin in Egypt^4,6^, Libya^34^, and Tunisia^28^. On the other hand, *P.* (*Phygraea*)* vesicularis* and *N. nicaisei* are widely distributed, and their palaeobiogeographic range will be further discussed below.

### Pycnodonte*** (Phygraea) vesicularis***

**Figure 9:**
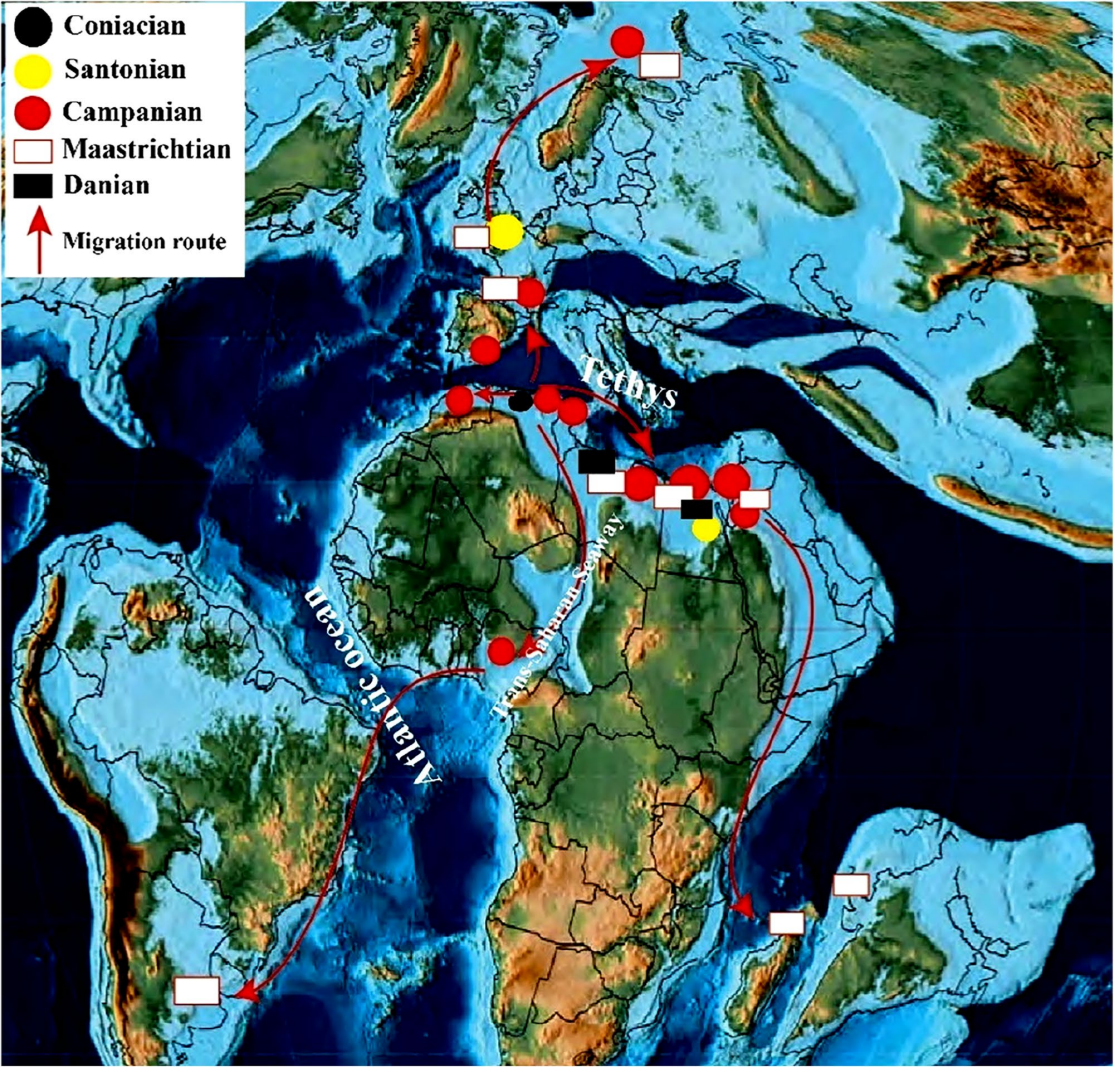
The palaeobiogeographic distributions of *Pycnodonte* (*Phygraea*) *vesicularis* (Lamarck, 1806) are illustrated in the map of early Campanian, sourced from Scotese^65^.

This species exhibits a broad temporal and paleogeographic distribution (Fig. 9), spanning from the Coniacian to the Danian^51,53^. This broad distribution emphasizes the species' adaptability to thrive in diverse environments across various geological ages. This eurytopic species first appeared in the southern part of the Tethys Sea in Tunisia. Thus, the Coniacian deposits in the Southern Tethys of Tunisia, as recorded by Schijfsma^63^, provide the oldest so far evidence of the oyster *Pycnodonte* (*Phygraea*) *vesicularis*. Following its first appearance in Tunisia, the species was recorded from Santonian deposits in the Southern Tethys (Egypt)^8,30,64^ and the Western Tethys (Belgium)^50^. The presence of this oyster species in geographically distant areas of the Tethys Sea during the Coniacian and Santonian periods suggests that *P. vesicularis* initially migrated from the Southern Tethys Sea in Tunisia towards the end of the Coniacian. It then expanded its range both eastward to the Santonian of Egypt's northern Tethys coastline and westward to the Western Tethys region, now situated in Belgium.

A second major migratory event for *P. vesicularis* occurred during the Santonian-Campanian transition, leading to its widespread distribution across the Tethys region during the Campanian (Fig. 9). It has been documented in various Southern Tethys regions, specifically in Tunisia^28^, Morocco^45^, Egypt^6,8,44,64,66^, Libya^52^, Palestine, and Jordan^33^. In addition, the species has been recorded in the Campanian of the Western Tethys, indicating its migration from Belgium to France^38,49^, Spain^67^, and Poland^46^. Additionally, this species has been recorded in the Nigerian post-Santonian deposits^68^. This West African occurrence suggests a probable marine connection between the Tethys regions and West Africa via the Trans-Sahara Epicontinental Sea during the Campanian—Maastrichtian period.

*P. vesicularis* underwent a significant third migration during the Campanian–Maastrichtian transition, leading to further expansion of its range. Maastrichtian records indicate its presence in the Southern Tethys deposits in Egypt^6,43,69^, Libya (according to Ref.^34^), and Jordan^33^, along with western Tethyan areas like France^49^, Belgium^70^, and Poland^46,47^. Notably, *P. vesicularis* extended its distribution to distant locations during the Maastrichtian, including Madagascar^71^, India^53^, and the Southern Atlantic coast in Argentina^61,72^. The Maastrichtian distribution suggests two concurrent secondary migrations that likely occurred during the Campanian–Maastrichtian transition. One trend involved a westward migration from the Trans-Sahara region to the South Atlantic Ocean province through a shallow sea passage. The other migration took place from the Southern Mediterranean eastward, reaching both India and Madagascar.

During the early Paleocene, *P. vesicularis* is recorded in two instances from the Southern Tethys, specifically Egypt^8,51,73^ and Libya^74^. Nonetheless, the absence of the species from Paleocene deposits across its previously extensive geographical range implies that the Southern Tethys population was less affected by the extinction event at the end of the Cretaceous period compared to populations in the North Tethys, Northeastern Tethys, and India. In addition, the Paleocene specimens from the Western Desert of Egypt exhibit notable differences from the Upper Cretaceous specimens, particularly in the presence of stronger commarginal ribs. These variations raise the necessity for re-evaluating this record and reassessing the species' taxonomic status.

Conversely, some authors have proposed that these early Paleocene records might originate from a reworked unit called the Bir Abu Minqar horizon^75–77^, which signifies the K/Pg boundary in Egypt's Western Desert. This suggests that *Pycnodonte (Phygraea) vesicularis* specimens recovered from these deposits may have been reworked from late Maastrichtian beds, rather than representing an authentic early Paleocene population in situ.

The present study agrees with the findings of Strougo^51^ and Hewaidy et al.^73^, supporting the statement that Pycnodonte (Phygraea) vesicularis, recorded from the Egyptian Western Desert, is indeed representative of the early Paleocene and not a result of reworking from the Maastrichtian deposits. This conclusion is drawn from the exceptional preservation of the recorded species and the conspicuousness of their specimen's ornamentation.

### Nicaisolopha*** nicaisei***

**Figure 10:**
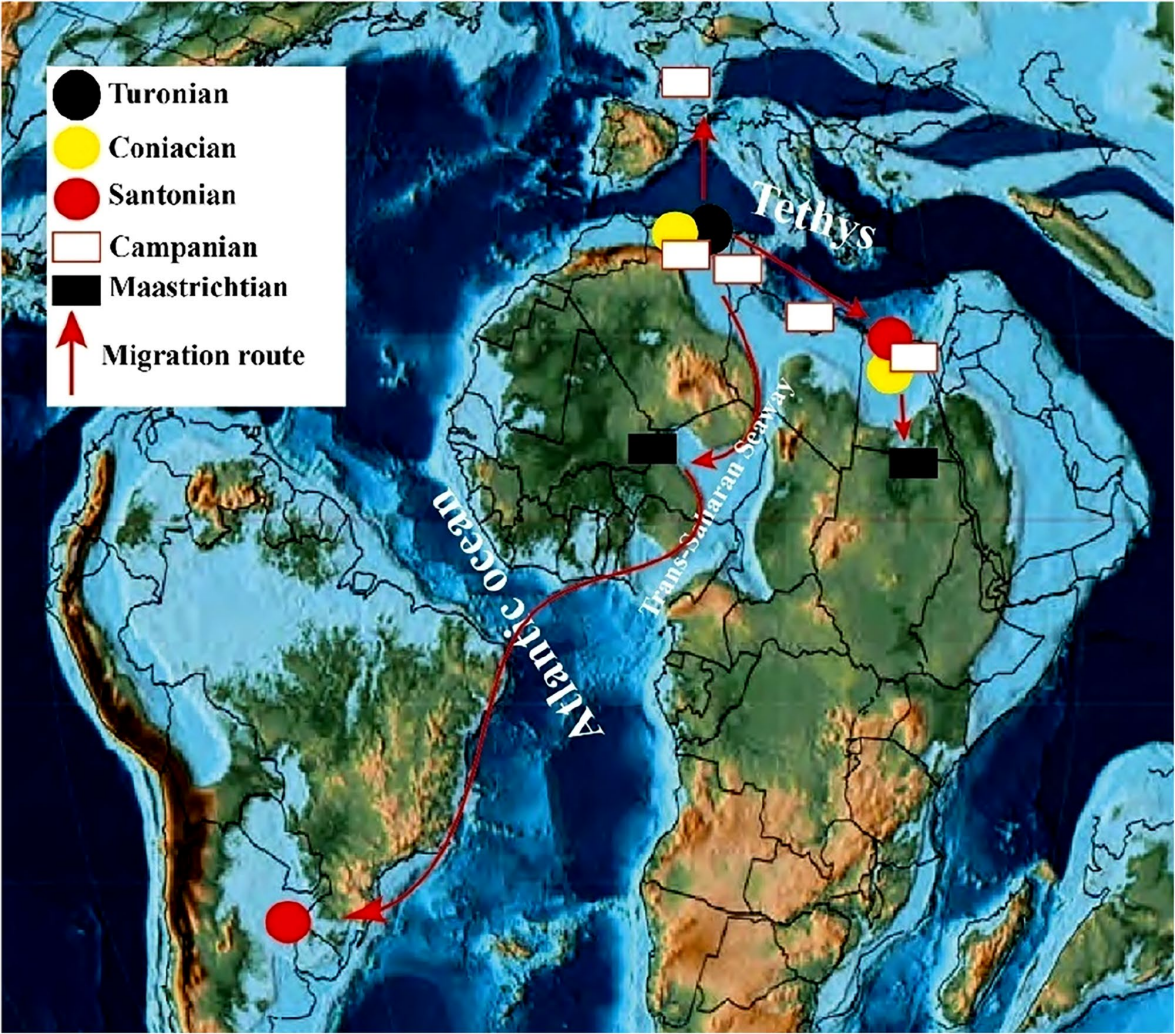
The palaeobiogeographic distributions of *Nicaisolopha nicaisei* (Coquand, 1862) are illustrated in the map of early Campanian, sourced from Scotese^65^.

The presence of *Nicaisolopha* Vialov, 1936^25^ in Egypt may be attributed to the potential migration of *Nicaisolopha lugubris* larvae from the southwestern United States of America to North Africa during the Turonian^5^. The genus has a range extending from the Late Cretaceous to the Quaternary. The earliest documented occurrence of the oyster *Nicaisolopha nicaisei* is from the Turonian deposits of Algeria in Southern Tethys (Fig. 10), as recorded by Benmansour^78^. The species has been recorded from the Coniacian deposits of the Southern Tethys in Egypt^4,5,9^ and Algeria^34^. A wealth of records has been found in the Santonian-Maastrichtian deposits of the Southern Tethys region. Notably, these include the Santonian deposits in Egypt^5,6,8^ and Algeria^34^, as well as the Campanian deposits in Egypt^4–6,30,32^, Algeria^26,28,34^, and Tunisia^28^. Moreover, it has been recorded in the uppermost Campanian–Maastrichtian deposits of Libya^34^. Evidence of migration from the Southern Tethys to Western Tethys is supported by its first European occurrence in the Campanian deposits of France^8^.

The persistence of Maastrichtian records in Egypt^4^ and Sudan^79^ indicates that the area encompassing Egypt and Sudan was submerged under a shallow epicontinental sea during the Maastrichtian age. Additionally, the oyster *Nicaisolopha nicaisei* has been recorded from the Maastrichtian deposits of the Trans-Saharan Seaway in Mali^80^. Beyond the Southern Tethys region, this oyster species has been recorded from the Coniacian-Santonian deposits of South America, specifically in Peru^35^.This finding raises the possibility of a potential migration route from the Southern Tethys, potentially through the Trans-Saharan Seaway.

In conclusion, during the Late Cretaceous period, the palaeogeography of the European, North African, and southwest Asian branches of the Tethyan Realm was interconnected via seaways, facilitating a broad geographic distribution of the Pycnodonteinae^3,34,81,82^, Flemingostreinae, and Liostreinae. Accordingly, several species within these subfamilies exhibited biogeographical distributions that spanned the basins of South Europe, North Africa, the Middle East, and SouthWest Asia. Moreover, the widespread distribution of the oysters *Nicaisolopha nicaisei* and *P.* (*Phygraea*)* vesicularis* can be attributed to the dispersal ability of their larvae through marine currents^3^. The changes in the Tethyan currents, shifting from West–East during the Albian-Cenomanian and Coniacian-Santonian periods to East–West during the Campanian–Maastrichtian, may be attributed to the tectonic events that occurred at the Santonian-Campanian transition^34^. Thus, the primary migration pattern of the studied oysters was likely from the Southern Tethys margin towards the East–West direction. The oysters identified in this study exhibit a Tethyan affinity and dominate two main provinces: the Southern Tethys province and the Western Tethys province.

## Conclusions

This study focuses on Campanian Tethyan oysters from the Central North Eastern Desert of Egypt, specifically examining their taxonomy, palaeobiogeography, and palaeoecology. Three oyster species were identified from two Campanian successions: *N. nicaisei*, *Pycnodonte* (*Phygraea*) *vesicularis*, and *Ambigostrea bretoni*. In this study, *N. tissoti* (Thomas and Peron, 1891) is considered a junior synonym of *Nicaisolopha nicaisei* (Coquand, 1862). The palaeobiogeographic analysis suggests the migration of the studied oysters from the Southern Tethys margin in an East–West direction. These oyster species were primarily linked to the Tethyan Realm and are prominent in the Southern Tethys and Western Tethys provinces. The macrofaunal contents examined in the study are categorized into two associations: *Nicaisolopha nicaisei* association and *Pycnodonte vesicularis* association. These macrofaunal associations suggest a transition towards deeper environments during the middle-late Campanian, shifting from shallow inner neritic to middle neritic habitats. Furthermore, species within the Pycnodonteinae subfamily tend to grow larger under eutrophic conditions, calm water environments, and nutrient-rich waters with high carbonate contents.
